# Aspiration biopsy versus dilatation and curettage for endometrial hyperplasia prior to hysterectomy

**DOI:** 10.1186/s13000-020-01065-0

**Published:** 2021-01-14

**Authors:** Woo Yeon Hwang, Dong Hoon Suh, Kidong Kim, Jae Hong No, Yong Beom Kim

**Affiliations:** Department of Obstetrics and Gynecology, Seoul National University Bundang Hospital, 82, Gumi-ro 173 Beongil, Bundang-gu, Seongnam-si, Gyeonggi-do 13620 Republic of Korea

**Keywords:** Endometrial hyperplasia, Dilatation and curettage, Hysterectomy, Biopsy

## Abstract

**Background:**

To compare the diagnostic accuracy of aspiration biopsy and dilatation and curettage (D&C) in patients diagnosed with endometrial hyperplasia prior to hysterectomy.

**Methods:**

We retrospectively reviewed medical records of 250 patients diagnosed with endometrial hyperplasia by endometrial sampling between July 2003 and March 2020. Endometrial sampling was performed by aspiration biopsy (*n* = 150) or D&C (*n* = 100), followed by hysterectomy within 6 months. Pathological findings of hysterectomy specimens of the two groups were compared to preoperative findings.

**Results:**

The overall diagnostic concordance between endometrial sampling specimen including D&C and aspiration biopsy, and hysterectomy specimen was 51.0% (51/100) and 41.3% (62/150), respectively. Patients whose preoperative specimen was obtained by D&C were upgraded less significantly than those who underwent aspiration biopsy (21.0% vs 36.7%; *P* = 0.008). In particular, significantly fewer patients were upgraded after D&C than after aspiration biopsy in hyperplasia without atypia (12.5% vs 29.0%; *P* = 0.028). In addition, when the final pathological upgrade rate to endometrial carcinoma was evaluated between the two methods of endometrial sampling, significantly fewer cases were noted after D&C than after aspiration biopsy (15.0% vs 27.3%; *P* = 0.022).

**Conclusions:**

In our study, D&C more accurately reflected the final diagnosis in patients with endometrial hyperplasia than aspiration biopsy based on the histological examination of hysterectomy specimens. When considering the management strategy for women with an endometrial hyperplasia diagnosis obtained by aspiration biopsy, physicians should consider the significant rate of upgraded diseases with this method of endometrial sampling.

## Introduction

Endometrial hyperplasia is a pathological condition characterized by abnormal proliferation of endometrial glands and stroma [1]. Endometrial hyperplasia, atypical endometrial hyperplasia in particular, is of clinical significance as it is considered to be a precursor lesion of endometrial cancer [2–4]. The accurate diagnosis of premalignant lesions and the exclusion of coexisting endometrial carcinomas are important aspects of the proper management of endometrial hyperplasia. The World Health Organization (WHO) 94 classifies endometrial hyperplasia into four groups based on glandular complexity and cytological nuclear atypia: simple hyperplasia without atypia, complex hyperplasia without atypia, simple atypical hyperplasia, and complex atypical hyperplasia [4].

There are several endometrial sampling methods used in practice to diagnose endometrial lesions, including endometrial hyperplasia. Among these, dilatation and curettage (D&C) has been one of the most widely used. However, it has become less favorable due to the added risk of anesthesia and complications. Endometrial aspiration biopsy has widely replaced D&C as it is easy to perform, safe, and convenient compared to D&C [5, 6].

However, there are no reliable data regarding the best method to diagnose endometrial hyperplasia. Numerous studies comparing the diagnostic accuracy of D&C and aspiration biopsy have concluded that aspiration biopsy is as accurate as D&C in the diagnosis of endometrial pathologies [5–8]. However, all these studies have included patients with various pathological findings such as proliferative endometrium, secretory endometrium, endometrial hyperplasia, atrophic endometrium, and endometrial carcinoma. In addition, they were limited by their small sample size, especially for evaluating endometrial hyperplasia. They included only a small number of patients with endometrial hyperplasia and fewer than 10 patients with atypical endometrial hyperplasia.

Therefore, in this study, we aimed to compare the diagnostic accuracy of aspiration biopsy and D&C in patients who were diagnosed with endometrial hyperplasia by one of these methods prior to hysterectomy.

## Methods

This study was approved by the Institutional Review Board of the Seoul National University Bundang Hospital (No. B-2007/627–101) and performed in accordance with the principles of the Declaration of Helsinki. The requirement for informed consent was waived.

This study was retrospectively performed between July 2003 and May 2020 at Seoul National University Bundang Hospital, a tertiary hospital in Korea.

We reviewed the medical records of 250 patients who had been diagnosed with endometrial hyperplasia either by aspiration biopsy or D&C and had subsequently undergone hysterectomy. The aspiration biopsy was performed without anesthesia using the Pipelle device. D&C was performed under sedation with fentanyl and midazolam or under monitored anesthesia care using a curette. Eligible patients included those diagnosed with hyperplasia without atypia (simple and complex) and atypical hyperplasia (simple and complex). The diagnoses of the endometrial sampling specimens were independently reviewed pathologically in our institution. The maximum time interval between endometrial sampling confirming endometrial hyperplasia and hysterectomy was 6 months. We excluded the patients who had a diagnosis of “endometrial hyperplasia, cannot rule out malignancy” and those with “insufficient tissue for pathological evaluation.” Additionally, those who underwent non-surgical management such as hormone therapy after the diagnosis of endometrial hyperplasia and prior to hysterectomy were excluded.

The pathological results of the preoperative endometrial sampling specimens were compared to those of the hysterectomy specimens. We evaluated the consistency of the pathological results between samples obtained by aspiration biopsy and by D&C. Results of “no residual disease,” “secretory endometrium,” and “proliferative endometrium” in postoperative specimen were considered normal.

The Student’s *t*-test and Mann-Whitney *U* test were performed to compare continuous variables. Pearson’s chi-squared test or Fisher’s exact test was performed to compare categorical variables. Kappa statistics were used to assess the agreement of the two endometrial sampling methods. κ values < 0 indicated no agreement, 0 to 0.20 slight, 0.21 to 0.40 fair, 0.41 to 0.60 moderate, 0.61 to 0.80 substantial, and 0.81 to 1 almost perfect agreement. All analyses were performed using SPSS software for Windows (version 25.0; SPSS Inc., Chicago, IL, USA). A *P* < 0.05 indicated statistical significance.

## Results

In this study, 250 patients were included. The overall patient characteristics are shown in Table 1. Of the 250 patients who had been diagnosed with endometrial hyperplasia, 100 were diagnosed by D&C and 150 by aspiration biopsy. There were 40 (16.0%) postmenopausal patients. In total, 154 (61.6%) patients underwent endometrial sampling due to abnormal uterine bleeding. The mean time between endometrial sampling and hysterectomy was 1.6 ± 1.0 months (mean ± SD). The histologic results by endometrial sampling showed 76 (30.4%) patients with simple hyperplasia without atypia, 42 (16.8%) with complex hyperplasia without atypia, 4 (1.6%) with simple atypical hyperplasia, and 128 (51.2%) with complex atypical hyperplasia. Final pathological results from hysterectomy confirmed 35 (14.0%) patients with normal endometrium, 68 (27.2%) with simple hyperplasia without atypia, 24 (9.6%) with complex hyperplasia without atypia, 3 (1.2%) with simple atypical hyperplasia, 64 (25.6%) with complex atypical hyperplasia, and 56 (22.4%) with carcinoma.

**Table 1:** Characteristics of the overall study population

Characteristics	n (%)
Age, years	49.0 ± 6.5
BMI^a^, kg/m^2^	24.8 ± 4.6
Parity	1.8 ± 0.7
Menopause	40 (16.0)
Diabetes	9 (3.6)
Hypertension	35 (14.0)
Tamoxifen	21 (8.4)
Abnormal uterine bleeding	154 (61.6)
EM sampling method	
D&C	100 (40.0)
Aspiration biopsy	150 (60.0)
Time interval between EM sampling & hysterectomy, months	1.6 ± 1.0
EM sampling pathology	
SH	76 (30.4)
CH	42 (16.8)
SAH	4 (1.6)
CAH	128 (51.2)
Hysterectomy pathology	
Normal	35 (14.0)
SH	68 (27.2)
CH	24 (9.6)
SAH	3 (1.2)
CAH	64 (25.6)
Carcinoma	56 (22.4)

Data are mean ± SD or n (%) unless otherwise specifiedik

^a^BMI data are missing for two patients

*BMI* Body mass index; *EM* Endometrial; *D&C* Dilatation and curettage; *SH* Simple hyperplasia without atypia; *CH* Complex hyperplasia without atypia; *SAH* Simple atypical hyperplasia; *CAH* Complex atypical hyperplasia

When the diagnostic concordance between D&C and hysterectomy was assessed, in a total of 100 patients, 51 (51.0%) had diagnostic concordance: 23 (23.0%) with simple hyperplasia without atypia, 9 (9.0%) with complex hyperplasia without atypia, and 19 (19.0%) with complex atypical hyperplasia (Table 2). In addition, when the diagnostic concordance between aspiration biopsy and hysterectomy was assessed, in a total of 150 patients, 62 (41.3%) had diagnostic concordance: 24 (16.0%) with simple hyperplasia without atypia, 6 (4.0%) with complex hyperplasia without atypia, 1 (0.7%) with simple atypical hyperplasia, and 31 (20.6%) with complex atypical hyperplasia (Table 3).

**Table 2:** Comparison of pathological results of D&C and hysterectomy

D&C	n (%)	Hysterectomy						Concordance to hysterectomy n (%)
		Normal	SH	CH	SAH	CAH	Carcinoma	
SH	36 (36.0)	9	23	1	0	2	1	23 (63.9)
CH	20 (20.0)	4	4	9	0	3	0	9 (45.0)
SAH	0 (0.0)	0	0	0	0	0	0	0 (0.0)
CAH	44 (44.0)	6	3	2	0	19	14	19 (31.8)
Total	100 (100.0)	0	23	9	0	19	0	51 (51.0) ^a^

Data are mean ± SD or n (%) unless otherwise specified

^a^Kappa value, 0.357 (Fair); diagnostic concordance 51.0% (51 out of 100 pairs); *P* < 0.001

*D&C* Dilatation and curettage; *SH* Simple hyperplasia without atypia; *CH* Complex hyperplasia without atypia; *SAH* Simple atypical hyperplasia; *CAH* Complex atypical hyperplasia

**Table 3:** Comparison of pathological results of aspiration biopsy and hysterectomy

Aspiration biopsy	n (%)	Hysterectomy						Concordance to hysterectomy n (%)
		Normal	SH	CH	SAH	CAH	Carcinoma	
SH	40 (26.7)	6	24	3	2	5	0	24 (60.0)
CH	22 (14.7)	2	6	6	0	4	4	6 (27.3)
SAH	4 (2.7)	1	1	0	1	0	1	1 (25.0)
CAH	84 (56.0)	7	7	3	0	31	36	31 (36.9)
Total	150 (100)	0	24	6	1	31	0	62 (41.3) ^a^

Data are mean ± standard deviation or n (%) unless otherwise specified

^a^Kappa value, 0.239 (Fair); diagnostic concordance 41.3% (62 out of 150 pairs); *P* < 0.001

*SH* Simple hyperplasia without atypia; *CH* Complex hyperplasia without atypia; *SAH* Simple atypical hyperplasia; *CAH* Complex atypical hyperplasia

We identified the upgrade rate of the final pathology based on the method of endometrial sampling. The final pathology was upgraded in significantly fewer cases after D&C than after aspiration biopsy (21.0% vs 36.7%; *P* = 0.008) (Table 4). Particularly, in patients with hyperplasia without atypia, upgrading was found in significantly fewer cases after D&C than after aspiration biopsy (12.5% vs 29.0%; *P* = 0.028). However, no significant difference in upgrading was observed between D&C and aspiration biopsy for atypical hyperplasia (31.8% vs 42.0%; *P* = 0.255).

**Table 4:** Comparison of postoperative pathological upgrade risks based on endometrial sampling methods

Endometrial sampling pathology	Upgrade on final pathology	Endometrial sampling method		*P*
		D&C (*n* = 100)	Aspiration biopsy (*n* = 150)	
Hyperplasia without atypia	No	49 (87.5)	44 (71.0)	0.028
	Yes	7 (12.5)	18 (29.0)	
Atypical hyperplasia	No	30 (68.2)	51 (58.0)	0.255
	Yes	14 (31.8)	37 (42.0)	
Total	No	79 (79.0)	95 (63.3)	0.008
	Yes	21 (21.0)	55 (36.7)	

Data are n (%) unless otherwise specified

*D&C* Dilatation and curettage

We also assessed the final pathological upgrade rate to endometrial carcinoma based on the method of endometrial sampling. Carcinoma was found in 56 (22.4%) of the hysterectomy specimens. The final pathology was upgraded to endometrial carcinoma in significantly fewer cases after D&C than after aspiration biopsy (15.0% vs 27.3%; *P* = 0.022) (Table 5). No significant difference in the upgrade rate was observed between the two endometrial sampling methods for hyperplasia without atypia and atypical hyperplasia (hyperplasia without atypia, 1.8% vs 6.5%; *P* = 0.368, atypical hyperplasia, 31.8% vs 42.0%; *P* = 0.255).

**Table 5:** Comparison of final pathological carcinoma risks based on endometrial sampling methods

Endometrial sampling pathology	Carcinoma on final pathology	Endometrial sampling method		*P*
		D&C (*n* = 100)	Aspiration biopsy (*n* = 150)	
Hyperplasia without atypia	No	55 (98.2)	58 (93.5)	0.368*
	Yes	1 (1.8)	4 (6.5)	
Atypical hyperplasia	No	30 (68.2)	51 (58.0)	0.255
	Yes	14 (31.8)	37 (42.0)	
Total	No	85 (85.0)	109 (72.7)	0.022
	Yes	15 (15.0)	41 (27.3)	

Data are n (%) unless otherwise specified

* Fisher’s exact test for hyperplasia without atypia

*D&C* Dilatation and curettage

## Discussion

Our comparison of the accuracy of aspiration biopsy and D&C in patients diagnosed with preoperative endometrial hyperplasia suggests that D&C is a more accurate endometrial sampling method than aspiration biopsy.

In patients diagnosed with endometrial hyperplasia, there are risks of hidden malignancy and the possibility of progression to endometrial carcinoma. The risk of progression is < 5% in women with endometrial hyperplasia without atypia but increases up to 30% in women with atypical endometrial hyperplasia [9]. Therefore, hysterectomy is recommended, particularly in patients with atypical endometrial hyperplasia [2]. However, in those who desire the preservation of their fertility or are unable to tolerate surgery due to coexisting medical conditions, conservative management is inevitable. In these patients, accurate diagnostic evaluation is particularly vital, as their final pathologic results might not be verified. In our study, 210 (84%) of the patients were premenopausal patients for whom special attention is required to exclude coexisting malignancy.

Various methods of endometrial sampling are used in either inpatient or outpatient settings as a basis to diagnose endometrial lesions. Past studies regarding the accuracy of D&C compared to that of endometrial biopsy in diagnosing endometrial hyperplasia show conflicting results. One prospective study investigated the comparable diagnostic value of D&C and biopsy [10]. Of the 70 patients, 55 and 45% of cases were diagnosed with complex atypical hyperplasia using endometrial biopsy and D&C, respectively. No difference in the incidence of coexisting cancer at hysterectomy was observed between the two methods (41% vs 45%; *P* > 0.05), but this study conclusion was limited by the small sample size. Another study comparing diagnostic accuracy between Pipelle endometrial sampling and D&C in women with postmenopausal bleeding presented that the Pipelle strategy has not only similar effect, but also has more cost-effective compared to the D&C [11]. The cost-effectiveness may be considered as an important part of clinical decision making between aspiration biopsy and D&C procedure. The study evaluating whether preoperative D&C lowers the risk of unexpected cancer at hysterectomy compared to biopsy alone and reported that D&C lowered the risk of unexpected carcinoma compared to endometrial biopsy in patients with complex atypical hyperplasia (30% vs 45%; *P* < 0.001) [12]. Dijkhuizen et al. performed a meta-analysis to assess the accuracy of endometrial sampling devices for detecting endometrial carcinoma and endometrial hyperplasia [13]. They concluded that aspiration biopsy with Pipelle is superior to other endometrial instruments with sensitivities of 99.6 and 91% for endometrial carcinoma and endometrial hyperplasia, respectively, and more than 98% specificity. However, this review primarily included studies that examined the final pathology using D&C. Of the 39 studies, only five included studies used hysterectomy as the reference for final pathology [14–18].

Compared to previous studies, our study has several strengths. First, we include a substantial number of patients diagnosed with endometrial hyperplasia, which was one of the limitations of previous studies [10]. Second, we evaluate the final pathology based on hysterectomy specimens to compare two endometrial sampling methods, which provides more accuracy than using D&C as a reference. Third, in our study, the patients with “insufficient tissue for pathological examination” were excluded to further clarify the results. One of the concerns about endometrial sampling is the sufficiency of sample specimen. In some studies, inadequate pathological specimens that may have affected the results were included or the adequacy of the specimens was not considered [8]. Lastly, we also excluded patients who received hormone therapy during the period between endometrial sampling and hysterectomy, as this treatment may significantly affect pathology.

One potential limitation of the current study is that we could not apply the updated endometrial hyperplasia classification. There are two classification systems of endometrial hyperplasia: the WHO94 classification, used in this study, and the endometrial intraepithelial neoplasm (EIN) classification. The current position statement of the American College of Obstetricians and Gynecologists and the Society of Gynecologic Oncology recommends using the EIN classification, which includes three categories: benign (benign endometrial hyperplasia), premalignant (EIN), and malignant (well-differentiated endometrial adenocarcinoma) [19]. The EIN classification is more likely to identify precancerous lesions than the WHO94 classification. However, as our pathology department has only recently converted to the new terminology, the old WHO94 classification was used in this study. The WHO94 classification remains the most commonly used and reported classification in existing literature [4]. Another limitation of the current study is that we did not evaluate the effect of endometrial thickness. Thickened endometrium is known to be associated with endometrial pathologies. However, this may have little impact on the result of our study. A previous study evaluating the effect of endometrial thickness between biopsy methods showed no statistically significant results [6]. The final limitation is that we did not compare various devices used for endometrial sampling. It is possible that differences in sampling devices may affect the accuracy of the results.

In conclusion, as a gold standard method of endometrial sampling, higher accuracy with final pathology is expected from D&C than from aspiration biopsy. For an accurate diagnosis of endometrial hyperplasia, D&C seems superior to aspiration biopsy. When considering management strategies for women with endometrial hyperplasia obtained from aspiration biopsy, physicians should take into account the considerable rate of upgraded diseases.

## Data Availability

All data generated or analyzed during this study are included in this published article.

## Abbreviations

D&C: Dilatation and CURETTAGE
WHO: World health Organization
EIN: Endometrial intraepithelial neoplasm
